# Deep Phenotyping of Chinese Electronic Health Records by Recognizing Linguistic Patterns of Phenotypic Narratives With a Sequence Motif Discovery Tool: Algorithm Development and Validation

**DOI:** 10.2196/37213

**Published:** 2022-06-03

**Authors:** Shicheng Li, Lizong Deng, Xu Zhang, Luming Chen, Tao Yang, Yifan Qi, Taijiao Jiang

**Affiliations:** 1 Institute of Systems Medicine Chinese Academy of Medical Sciences & Peking Union Medical College Beijing China; 2 Suzhou Institute of Systems Medicine Suzhou China; 3 Guangzhou Laboratory Guangzhou China; 4 Guangzhou Medical University Guangzhou China

**Keywords:** deep phenotyping, Chinese EHRs, linguistic pattern, motif discovery, pattern recognition

## Abstract

**Background:**

Phenotype information in electronic health records (EHRs) is mainly recorded in unstructured free text, which cannot be directly used for clinical research. EHR-based deep-phenotyping methods can structure phenotype information in EHRs with high fidelity, making it the focus of medical informatics. However, developing a deep-phenotyping method for non-English EHRs (ie, Chinese EHRs) is challenging. Although numerous EHR resources exist in China, fine-grained annotation data that are suitable for developing deep-phenotyping methods are limited. It is challenging to develop a deep-phenotyping method for Chinese EHRs in such a low-resource scenario.

**Objective:**

In this study, we aimed to develop a deep-phenotyping method with good generalization ability for Chinese EHRs based on limited fine-grained annotation data.

**Methods:**

The core of the methodology was to identify linguistic patterns of phenotype descriptions in Chinese EHRs with a sequence motif discovery tool and perform deep phenotyping of Chinese EHRs by recognizing linguistic patterns in free text. Specifically, 1000 Chinese EHRs were manually annotated based on a fine-grained information model, PhenoSSU (Semantic Structured Unit of Phenotypes). The annotation data set was randomly divided into a training set (n=700, 70%) and a testing set (n=300, 30%). The process for mining linguistic patterns was divided into three steps. First, free text in the training set was encoded as single-letter sequences (P: phenotype, A: attribute). Second, a biological sequence analysis tool—MEME (Multiple Expectation Maximums for Motif Elicitation)—was used to identify motifs in the single-letter sequences. Finally, the identified motifs were reduced to a series of regular expressions representing linguistic patterns of PhenoSSU instances in Chinese EHRs. Based on the discovered linguistic patterns, we developed a deep-phenotyping method for Chinese EHRs, including a deep learning–based method for named entity recognition and a pattern recognition–based method for attribute prediction.

**Results:**

In total, 51 sequence motifs with statistical significance were mined from 700 Chinese EHRs in the training set and were combined into six regular expressions. It was found that these six regular expressions could be learned from a mean of 134 (SD 9.7) annotated EHRs in the training set. The deep-phenotyping algorithm for Chinese EHRs could recognize PhenoSSU instances with an overall accuracy of 0.844 on the test set. For the subtask of entity recognition, the algorithm achieved an F1 score of 0.898 with the Bidirectional Encoder Representations from Transformers–bidirectional long short-term memory and conditional random field model; for the subtask of attribute prediction, the algorithm achieved a weighted accuracy of 0.940 with the linguistic pattern–based method.

**Conclusions:**

We developed a simple but effective strategy to perform deep phenotyping of Chinese EHRs with limited fine-grained annotation data. Our work will promote the second use of Chinese EHRs and give inspiration to other non–English-speaking countries.

## Introduction

Currently, electronic health records (EHRs) are increasingly becoming an important source for clinical data mining and analysis [1]. Phenotype information that describes patients’ clinical manifestations is one of the most valuable clinical information types in EHRs [2]. However, phenotype information in EHRs is mainly recorded in free text, which computers have difficulty using directly [3,4]. Therefore, it is important to develop natural language processing (NLP) technology to effectively structure phenotype information in free text. The NLP technology for structuring phenotype information in EHRs is called EHR-based phenotyping [5].

There are two key factors involved in EHR-based phenotyping [6]. The first factor is the development of an information model that can define the normalized target of phenotyping [7]. The second factor is the development of a phenotyping algorithm that can process phenotype information into a predefined information model [8]. In recent years, the focus of EHR-based phenotyping methods has shifted from the coarse-grained level to the fine-grained level [9,10]. Compared with coarse-grained phenotyping, fine-grained phenotyping can capture more phenotype details, including the phenotype concept and its associated attributes [11]. For example, in the free-text description “a sudden severe pain in the right-lower abdomen,” a fine-grained deep-phenotyping method not only considers the phenotype “pain” but also its associated attributes of body location (“abdomen”), temporal pattern (“acute”), and severity (“severe”). EHR-based phenotyping that can characterize phenotype details at a fine-grained level is called EHR-based deep phenotyping [12].

Deep-phenotyping methods can characterize phenotype information in a high-fidelity way, which can potentially improve the accuracy of EHR-based applications, such as disease diagnosis and treatment [13]. Hence, deep phenotyping has become the focus of medical informatics. In recent years, a series of deep-phenotyping methods for English EHRs have been developed. For example, Peterson et al [14] used the MetaMap tool [15] to recognize phenotype concepts in EHRs, along with a neural network model to predict attribute values associated with phenotypes. They finally characterized English EHRs with the Fast Healthcare Interoperability Resources (FHIR) model [16]. Xu et al [17] developed a bidirectional long short-term memory and conditional random field (Bi-LSTM-CRF) model to recognize phenotype concepts in EHRs, together with a machine learning method to predict attribute values, and finally represented the phenotype information in English EHRs with the clinical element model (CEM) [18]. Compared to the progress of deep-phenotyping English EHRs, the method for deep-phenotyping Chinese EHRs is still in its infancy. Regarding the existence of linguistic differences, the established strategies [14,17,19,20] for deep-phenotyping English EHRs cannot be directly used for Chinese EHRs. Moreover, developing a deep-phenotyping algorithm requires fine-grained annotation data. However, it is hard to obtain a large volume of annotation data because of the high annotation cost. This means that the development of a deep-phenotyping algorithm for Chinese EHRs suffers from the challenge of low-resource scenarios [8], so it is worth considering how to develop a generalized algorithm for deep-phenotyping Chinese EHRs with limited fine-grained annotated data.

In previous work, we developed a fine-grained information model named PhenoSSU (Semantic Structured Unit of Phenotypes) [21], which can accurately characterize phenotype information from medical guidelines with 12 attributes from SNOMED CT (Systematized Nomenclature of Medicine–Clinical Terms). To explore an effective strategy for deep-phenotyping Chinese EHRs, we tried to annotate some Chinese EHRs with the PhenoSSU model. During the annotation process, some linguistic patterns of PhenoSSU instances were found to frequently occur in the free text of Chinese EHRs. For example, there is a linguistic pattern of “attribute + attribute + attribute + phenotype” in a given Chinese sentence “患者反复出现(attribute)剧烈(attribute)腹部(attribute)疼痛(phenotype)” (English translation: “patients with repeated severe abdominal pain”). If the linguistic patterns of PhenoSSU instances could be effectively learned from the corpus of Chinese EHRs, it would be possible to perform deep phenotyping of Chinese EHRs by scanning linguistic patterns of PhenoSSU instances. Therefore, how to effectively learn linguistic patterns of PhenoSSU instances from the corpus of Chinese EHRs has become an important question.

Although linguistic patterns of PhenoSSU instances can be observed and summarized manually, this is a time-consuming process that depends on experienced experts. In the field of linguistic pattern mining, the Apriori-based method is one of the most representative algorithms, which was based on the principle of frequency counts of keyword occurrences [22]. The Apriori algorithm is well suited to simple linguistic pattern mining based on word co-occurrence. For example, a recent study used the Apriori algorithm to learn linguistic patterns of cyberbullying behaviors in a social networking service [23]. When two keywords co-occur frequently, they are considered to constitute a potential linguistic pattern, such as the co-occurrence of “foolish” and “abuse.” However, the linguistic patterns of the PhenoSSU instances are more complicated. Thus, Apriori-based methods are not competent at mining linguistic patterns of PhenoSSU instances because they cannot handle the co-occurrence of a phenotype and several attribute values simultaneously. Inspired by the work of Ofer et al [24], which considered biological sequences, such as DNA sequences, as human language and used advanced NLP tools to tackle biological tasks, we aimed to model Chinese EHRs as DNA-like sequences and mine linguistic patterns with advanced bioinformatics tools. In a recent review, Castellana et al [25] surveyed 16 classic DNA motif discovery tools and evaluated their ability to discover sequence motifs nested in 29 simulated sequence data sets. The MEME (Multiple Expectation Maximums for Motif Elicitation) motif discovery tool performed best among the 16 classic DNA motif discovery tools. In this study, we characterized phenotypes as “P” and attributes as “A” to transform the free text into a single-letter sequence that could be analyzed with the MEME motif discovery tool. The sequence motifs discovered in this single-letter sequence could be viewed as linguistic patterns of PhenoSSU instances in Chinese EHRs. Based on the linguistic patterns discovered in EHRs, we could identify PhenoSSU instances by recognizing linguistic patterns in free text. To summarize, the task of deep phenotyping of Chinese EHRs could be converted into two consecutive steps of sequence motif discovery and linguistic pattern recognition.

Following this idea, we aimed to identify linguistic patterns of PhenoSSU instances in Chinese EHRs with a biological sequence motif discovery tool and develop a deep-phenotyping algorithm for Chinese EHRs by scanning linguistic patterns in free text. The rest of this paper is organized as follows. The first section introduces the composition of the PhenoSSU model and its common linguistic patterns in free text. The second section introduces the method for using a biological sequence motif discovery tool to learn linguistic patterns from the corpus of Chinese EHRs. The third section introduces the method for recognizing PhenoSSU instances from Chinese EHRs based on linguistic patterns. The final section introduces a case study to illustrate the potential application of the deep-phenotyping algorithm. Although the deep-phenotyping algorithm developed in this study can only deal with Chinese EHRs, the underlying methodology can also be illuminating for other non–English-speaking countries.

## Methods

### Overview

**Figure 1:**
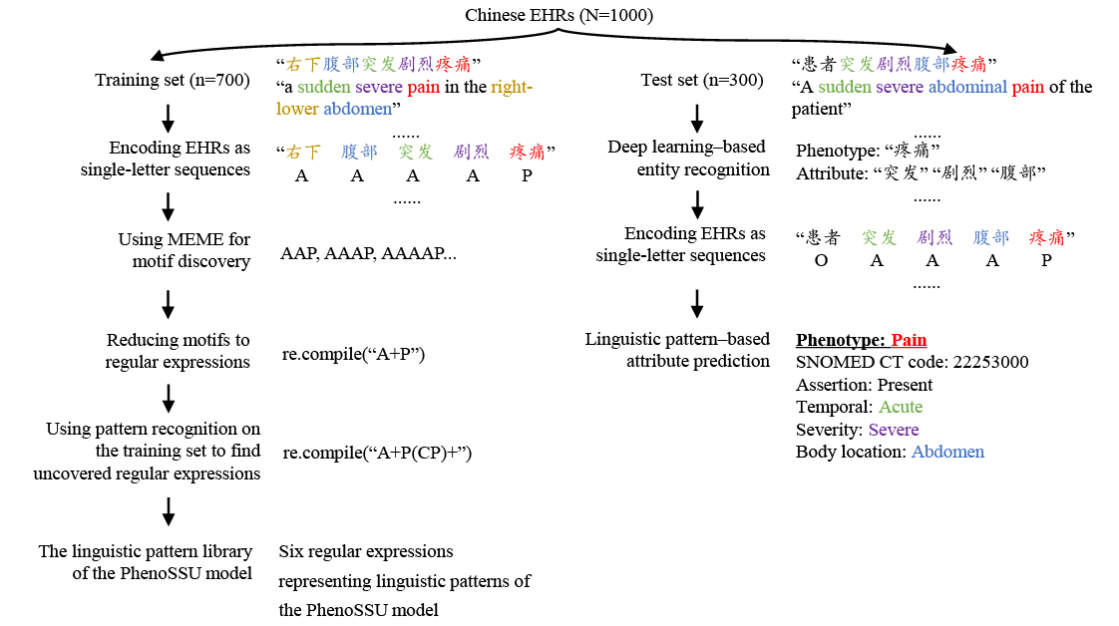
The pipeline for the linguistic pattern–learning method. A: attribute; C: punctuation; EHR: electronic health record; MEME: Multiple Expectation Maximums for Motif Elicitation; O: other information; P: phenotype; PhenoSSU: Semantic Structured Unit of Phenotypes; re.compile: a Python method used to compile a regular expression pattern; SNOMED CT: Systematized Nomenclature of Medicine–Clinical Terms.

In this study, a data-driven approach was proposed for learning linguistic patterns from Chinese EHRs. By using a pipeline of encoding the training set as a single-letter sequence and analyzing the sequence with the MEME motif discovery tool, we learned of six regular expressions and then introduced them into our pattern recognition–based algorithm for attribute prediction. The whole pipeline for the linguistic pattern–learning method is shown in Figure 1.

### The Design of the PhenoSSU Model for Representing Phenotype Information in Chinese EHRs

PhenoSSU is essentially an entity-attribute-value model consisting of phenotype terms along with standardized attributes from SNOMED CT. Compared with two commonly used information models named CEM and FHIR, the PhenoSSU model is more suitable for the task of deep phenotyping for two reasons. First, it has been shown that the PhenoSSU model is better at representing phenotype information in medical text than CEM and FHIR models [21]. Second, the PhenoSSU model puts more focus on characterizing phenotype traits with standardized attribute and value sets; as well, the attribute and value sets of the PhenoSSU model are easier to adjust according to the study-specific corpus.

To develop a fine-grained annotated corpus, 1000 Chinese EHRs of respiratory system diseases were manually annotated based on the PhenoSSU model, whose design was based on infectious diseases with a large proportion of respiratory diseases [21]. These 1000 Chinese EHRs were obtained from the EHR database of the Iiyi website [26]; all of the patients’ private information in these EHRs have been masked by the Iiyi website.

During manual annotation, we optimized the attributes included in the PhenoSSU model to make them suitable for Chinese EHRs. The optimized PhenoSSU model contained 10 attributes, which could be further divided into two subtypes: (1) attributes for phrase-based phenotypes, such as “heavy cough” or “fever,” including assertion, severity, temporal pattern, laterality, spatial pattern, quadrant pattern, and body location, and (2) attributes for logic-based phenotypes, such as “WBC [white blood cell] 12.5 × 10^9^/L,” including specimen, analyte, and abnormality. The composition of the PhenoSSU model is shown in Figure S1 and Table S1 in Multimedia Appendix 1, as well as the definitions, typical values, and SNOMED CT codes of attributes included in the model.

**Figure 2:**
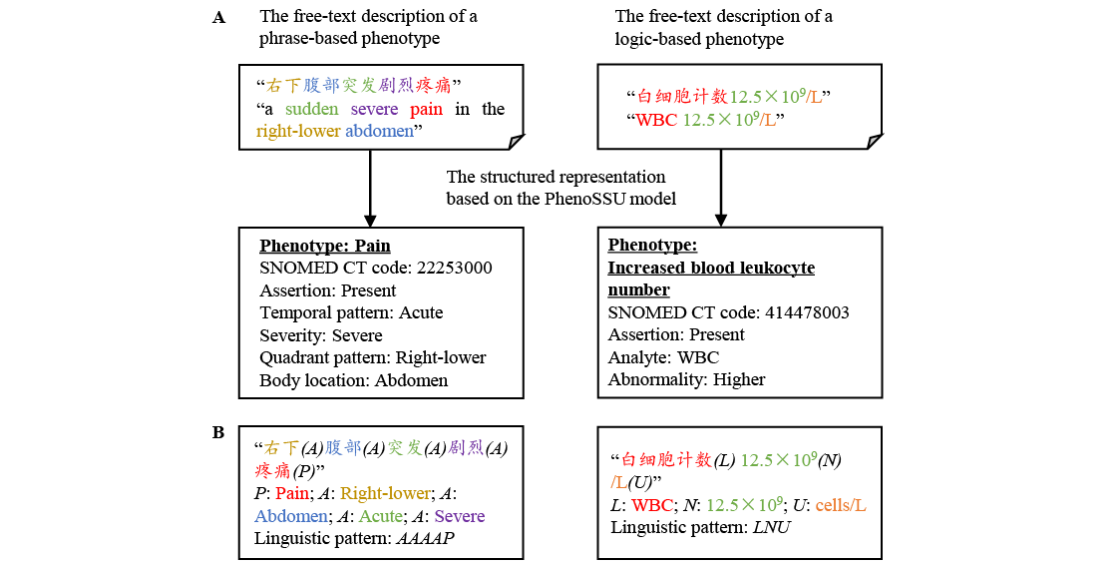
Free-text phenotype descriptions and linguistic patterns. A. Examples of structuring free text by the PhenoSSU model. B. Examples of linguistic patterns in free text. A: attribute; L: analyte; N: number; P: pain; PhenoSSU: Semantic Structured Unit of Phenotypes; U: unit; WBC: white blood cell.

The phenotype information in free text could be structurally represented by the PhenoSSU model. For example, the description “a sudden severe pain in the right-lower abdomen” could be represented as a PhenoSSU instance consisting of the phenotype concept “pain,” the assertion attribute “present,” the temporal pattern attribute “acute,” the severity attribute “severe,” the quadrant pattern attribute “right-lower,” and the body location attribute “abdomen.” Meanwhile, logic-based phenotypes (ie, qualitative and quantitative test results) were also included in the PhenoSSU model. For example, “WBC 12.5 × 10^9^/L” could be represented as a PhenoSSU instance consisting of the analyte “WBC” and the abnormality attribute “abnormality: higher,” which was combined and normalized as a concept of the “increased blood leukocyte number (414478003)” in SNOMED CT (Figure 2, A). The relevant knowledge came from our previous study, LATTE (transforming lab test results) [27], which was integrated into this work, including sample sources, analyte names, and reference ranges for 1098 laboratory tests. Detailed information about the knowledge base is shown in Figures S2 and S3 in Multimedia Appendix 1.

Based on the annotation guideline of the PhenoSSU model in our previous work, two Chinese authors with medical backgrounds (LC and SL) manually annotated these medical records independently. Annotations were made on the brat rapid annotation tool platform [28]. The initial annotating agreement measured with the Cohen κ statistic was 0.851. All inconsistent annotations were decided by the project supervisor (TJ).

During annotation, we found some linguistic patterns of PhenoSSU instances in the EHR text. For example, the description of a phrase-based phenotype, “右下腹部突发剧烈疼痛” (English translation: “a sudden severe pain in the right-lower abdomen”), could be summarized as “attribute (right-lower) + attribute (abdomen) + attribute (acute) + attribute (severe) + phenotype (pain).” Similarly, the description of logic-based phenotypes had common linguistic patterns in free text, such as “analyte (WBC) + number (12.5 × 10^9^) + unit (cells/ L)” (Figure 2, B). If we can mine linguistic patterns of PhenoSSU instances from Chinese EHRs, it would be possible to develop pattern recognition–based deep phenotyping.

### Learning Linguistic Patterns of PhenoSSU Instances From Chinese EHRs Using MEME: Workflow

#### Overview

**Figure 3:**
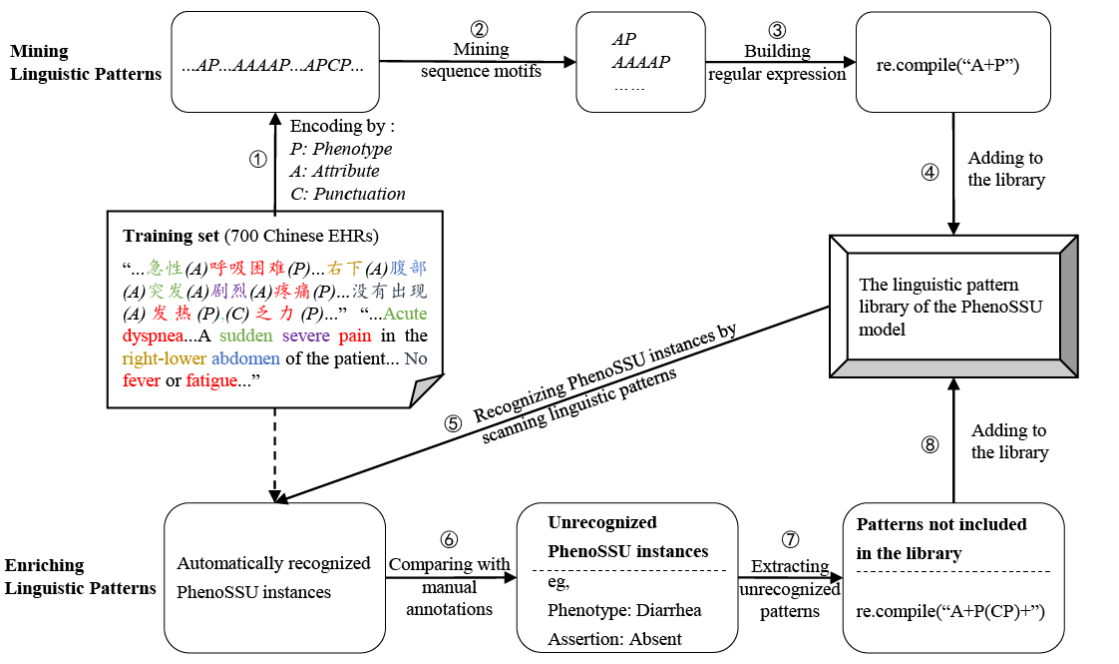
The workflow of learning linguistic patterns of the PhenoSSU model from the corpus of Chinese electronic health records (EHRs). PhenoSSU: Semantic Structured Unit of Phenotypes; re.compile: a Python method used to compile a regular expression pattern.

In order to learn linguistic patterns of PhenoSSU instances from the Chinese EHR corpus, 1000 annotated Chinese EHRs in the study were divided into a training set (n=700, 70%) and test set (n=300, 30%). The workflow of linguistic pattern mining is shown in Figure 3, which includes two stages: pattern discovery and pattern enrichment. In the stage of linguistic pattern discovery, we used the MEME motif discovery tool, which solves the problem of motif mining with a maximum likelihood method [29] to obtain seed linguistic patterns of PhenoSSU instances. In the stage of linguistic pattern enrichment, a semiautomatic method was developed to check and fill linguistic pattern gaps. Through pattern discovery and enrichment, we built a linguistic pattern library of PhenoSSU instances.

#### Stage 1: Linguistic Pattern Discovery

First, free text in the training set was encoded into single-letter sequences. To represent EHRs as the input of the MEME motif discovery tool, we encoded them as single-letter sequences with the following criteria: the phenotype (ie, “fever” and “cough”) was encoded as “P” and the attribute (ie, “severe”) was encoded as “A.” In the description of phrase-based phenotypes, “P” and “A” could be directly recognized in the original text. However, to calculate the abnormality of a logic-based phenotype, we need to combine the specimen (“S”), analyte (“L”), number (“N”), and unit (“U”). Specifically, the source of laboratory examination (ie, “blood” and “urine”) was encoded as “S,” the analyte (ie, “leukocyte”) was encoded as “L,” the number was encoded as “N” (ie, “37”), and the unit (ie, “°C”) was encoded as “U.” Meanwhile, the punctuation (ie, a comma) was encoded as “C,” and other information was encoded as “O.” In this study, EHRs were encoded using the FlashText tool, a tool for string-based concept recognition and replacement [30]. FlashText can find and replace keywords based on the trie dictionary data structure, which is 82 times faster than regular expressions. Because of its efficiency in processing text, we chose the FlashText tool for encoding text as single-letter sequences. Note that FlashText can retain the index of the strings in the original text. For example, the free-text description “患者主诉(O)急性(A)呼吸困难(P)...右下(A)腹部(A)突发(A)剧烈(A)疼痛(P)...没有出现(A)发热(P), (C)乏力(P)” (English translation: “Patient complained of acute dyspnea...A sudden severe pain in the right-lower abdomen...No fever and fatigue”) could be encoded as “AP...AAAAP...APCP*.*” During this stage, we finally obtained single-letter sequences from whole EHRs in the training set.

Second, the MEME motif discovery tool was used to mine motifs in the single-letter sequence. The pipeline of MEME motif discovery is composed of three steps: finding starting points, maximizing the likelihood expectation, and scoring the discovered motifs.

The input was a set of unaligned sequences, and the output was a list of probable motifs. The statistical significance of the motifs in MEME was evaluated by the *E* value, which is based on the log-likelihood ratio. The settings of the MEME motif discovery tool were optimized as follows:

1. Motif discovery mode: classic mode. In classic mode, only one sequence needs to be provided. The algorithm will find the repeated sequence fragments in the sequence by likelihood ranking.
2. Select the site distribution: any number of repetitions. This option means selecting motifs that occur repeatedly.
3. How wide can motifs be: from 2 to 30. This number is the width (ie, characters in the sequence pattern) of a single motif. MEME can choose an optimal width of each motif individually by using a heuristic function. In the process, there were some motifs containing “O” (ie, other information), which was irrelevant to phenotype descriptions. Therefore, we separated out the motifs with the letter “O” to generate sequence segments that may represent linguistic patterns of PhenoSSU instances.

Third, we built regular expressions based on the discovered motifs. To make the motifs available in our algorithm, regular expressions were built. For example, we built a regular expression “A+P” based on motifs or sequence segments generated from motifs like “AP,” “AAP,” “AAAP,” and “AAAAP.”

#### Stage 2: Linguistic Pattern Enrichment

**Figure 4:**
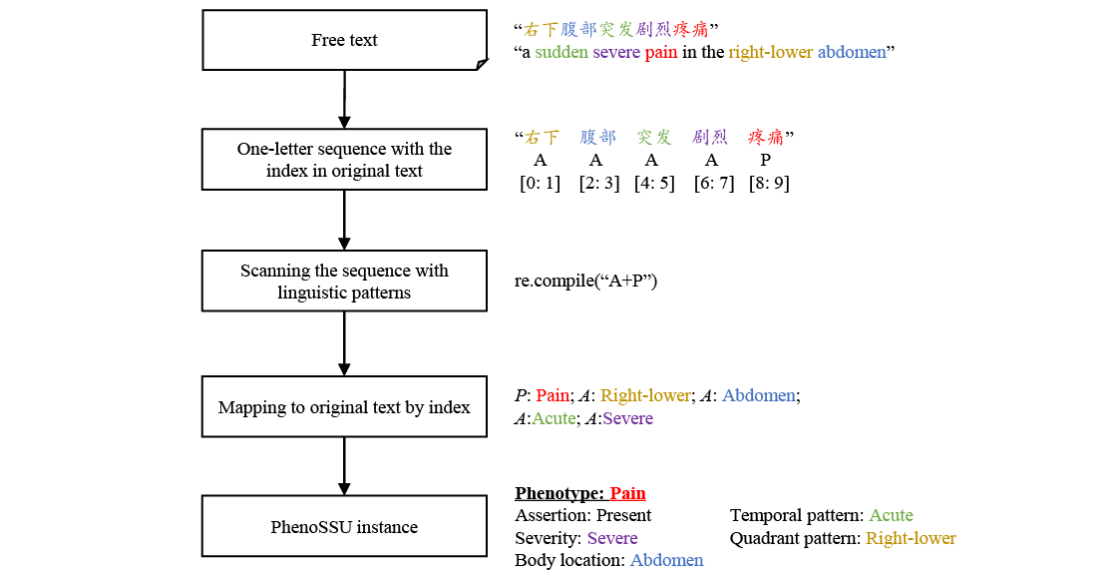
The workflow of recognizing PhenoSSU instances from free text via linguistic pattern recognition. The numbers within the square brackets represent the position indexes of single letters in the original text. A: attribute; P: phenotype; PhenoSSU: Semantic Structured Unit of Phenotypes; re.compile: a Python method used to compile a regular expression pattern.

In this stage, a linguistic pattern recognition–based method was first used to automatically recognize PhenoSSU instances from Chinese EHRs in the training set. The workflow of linguistic pattern recognition is shown in Figure 4, which includes the following steps:

1. Encoding text as single-letter sequences. For example, the description “右下腹部突发剧烈疼痛” (English translation: “a sudden severe pain in the right-lower abdomen”) was encoded as the single-letter sequence “AAAAP.” The FlashText tool could record the position index of Chinese characters in every single letter, making it possible to map single letters to the original text. An example of the position index recording is shown in Figure S4 in Multimedia Appendix 1.
2. Scanning the single-letter sequence with the linguistic patterns. In this case, “AAAAP” matched the linguistic pattern “A + P” perfectly, meaning that the four attributes were associated with the phenotype.
3. Mapping these letters to the original text by index. A: right-lower; A: abdomen; A: acute; A: severe; P: pain.
4. Filling phenotypes and associated attributes in the PhenoSSU model. Finally, the description “右下腹部突发剧烈疼痛” could be transformed into a PhenoSSU instance consisting of the phenotype “pain,” the assertion attribute “present,” the temporal pattern attribute “acute,” the severity attribute “severe,” the quadrant pattern attribute “right-lower,” and the body location attribute “abdomen.”

Based on the above steps, we discovered the unrecognized PhenoSSU instances by comparing the automatically recognized instances with manual annotation. For example, the description “没有出现(A)发热(P),(C)乏力(P)” (English translation: “No fever or fatigue”) could be encoded as “APCP,” in which “AP” matched the regular expression (“A + P”) in our pattern library. By mapping to the original text, “没有出现发热，乏力” was transformed into a PhenoSSU instance consisting of the phenotype “fever” and the assertion attribute “absent.” However, “absent” was also the attribute of the phenotypes “diarrhea” and “weight loss,” which were not recognized by the algorithm.

Finally, to check why these PhenoSSU instances were not recognized, all of them were encoded as single-letter sequences, which could be scanned with linguistic patterns. If no pattern matched, we collected such sequences to build new regular expressions and add them to the linguistic pattern library. In this example, sequences such as “APCPCP” were enriched into a regular expression “(A + P (CP) +).”

### Recognizing PhenoSSU Instances From Chinese EHRs: Workflow

The recognition of PhenoSSU instances could be divided into two subtasks: entity recognition and attribute prediction. To find the best strategy for the two tasks, it was essential to compare our proposed method with current state-of-the-art methods.

The first subtask was entity recognition, which aimed to recognize the text spans corresponding to phenotype and attribute entities. For the subtask of named entity recognition from Chinese EHRs, the Bidirectional Encoder Representations from Transformers (BERT)–Bi-LSTM-CRF model has proven its effectiveness in the CCKS (China Conference on Knowledge Graph and Semantic Computing) 2018 Task 1: Named Entity Recognition in Chinese Electronic Medical Records, which achieved the best F1 score of 91.43 [31]. Therefore, we compared algorithm performances of the BERT-Bi-LSTM-CRF model and the classic dictionary-based method in this study. The parameters of the BERT model were trained with the Kashgari package in Python (version 3.6.1; Python Software Foundation). In the dictionary-based method, the knowledge base of phenotypes was derived from the Chinese translations of the International Classification of Diseases, 10th Revision and 11th Revision, and the Human Phenotype Ontology (details in Table S2 in Multimedia Appendix 1). Further, the knowledge base of attribute trigger words was from the annotation of the training set. Entity recognition, combined with the other coding rules, was applied to encode free text as single-letter sequences, which would be used in the subsequent attribute prediction subtask.

The phenotype’s attribute recognition was the second subtask, which aimed to predict appropriate values for the 10 attributes in the PhenoSSU model. The encoded single-letter sequences from the free text and the developed pattern recognition algorithm in the first subtask were used for attribute prediction. For the subtask of attribute prediction, we did not compare our pattern recognition algorithm with currently existing methods because the PhenoSSU model is a relatively new information model, and algorithms for deep-phenotyping Chinese EHRs based on the PhenoSSU model are very scarce. However, we have referred to state-of-the-art algorithms for deep-phenotyping English EHRs. For example, our previous work showed that the support vector machine (SVM)–based model performed best in the task of deep phenotyping of English clinical guidelines. That is why the SVM model was compared with the linguistic pattern–based method in this study. Three features were used in the SVM model: (1) the distance between phenotype and attribute words, (2) the number of pauses between phenotype and attribute words, and (3) the characteristics of attribute words (eg, some attribute words were only on the left side of phenotype words). The SVM model was built with the scikit-learn package (version 1.1.0) in Python. The parameter tuning of the SVM model was based on a hybrid search strategy. In this study, we did not use deep learning–based methods, because our previous work showed that they were not good at recognizing PhenoSSU instances, owing to the lack of training samples [21].

### Evaluation of Algorithm Performance for Recognizing PhenoSSU Instances

To evaluate the algorithm’s performance for recognizing PhenoSSU instances, we used the evaluation metrics in SemEval (Semantic Evaluation) 2015 Task 14: Analysis of Clinical Text [32].

In the subtask of entity recognition, the F1 score was taken as the evaluation metric. When a predicted entity word entirely coincided with a gold-standard text span, it was considered as a true positive. The precision metric was calculated as the fraction of correctly predicted entities among all entities identified by the algorithm, and the recall metric was calculated as the fraction of correctly predicted entities among all entities identified by the annotators. The F1 score was calculated as the harmonic mean of precision and recall.

In the subtask of attribute prediction, the average accuracy and weighted average accuracy were taken as the evaluation metrics because the weighted average accuracy thoroughly considered the distribution of each attribute value in the corpus, which could better evaluate those attribute values with little distribution.

For the evaluation at the PhenoSSU-instance level, we used the combination of the F1 score for entity recognition and weighted average for attribute prediction. A PhenoSSU instance was considered correct when the phenotype and associated attribute values annotated by the algorithm were the same as the corresponding PhenoSSU instance annotated by experts.

### Ethical Considerations

The 1000 Chinese EHRs of respiratory system diseases used in this study were obtained from the EHR database of the Iiyi website [26]. No ethics approval was needed because the data from downloaded EHRs, including patients’ private information, were all masked by the Iiyi website.

## Results

### Linguistic Patterns of PhenoSSU Instances Learned From Chinese EHRs

A total of 51 sequence motifs were discovered from the Chinese EHRs in the training set (details are shown in Figure S5 in Multimedia Appendix 1). Based on the 51 motifs, we built six regular expressions (Table 1), namely linguistic patterns of the PhenoSSU instances in the Chinese EHRs. Among the regular expressions of phrase-based phenotypes, “AP +” appeared most frequently. The most common description of this regular expression was “absent” plus phenotypes, which could be used for differential diagnosis in clinical practice. The second frequent regular expression was “A + P,” which usually corresponded to a detailed description of phenotypes, such as “body location + severity + phenotype.” There were also complex linguistic patterns to be generalized as “A × PC × A +,” for example, “严重(A)咳嗽(P)，(C)呈持续性(A)” (ie, severe cough, consistently). Among the regular expressions of logic-based phenotypes, the most typical was “S × LNU,” such as the description “WBC 12 × 10^9^/L.” There were also linguistic patterns that directly interpreted laboratory examination results: “S × LR [results of laboratory examination],” such as “血糖升高” (ie, high blood glucose). The above results suggest that there are inherent linguistic patterns in Chinese EHRs. The detailed frequency of linguistic patterns is shown in Table S3 in Multimedia Appendix 1.

In this study, six regular expressions were learned from 700 Chinese EHRs in the training set. However, the size of the training set could be smaller than 700 in order to build the six regular expressions. To explore the potential smallest size of the training set, we conducted an experiment to explore the minimum number of EHRs that could match all six regular expressions. In the experiment, we randomly selected EHRs from the training set with stepwise increased data size, which were scanned with the six regular expressions. When all six regular expressions could be matched, that data size was recorded. This process was repeated 1000 times to calculate the mean and SD of the EHR sums that covered the six regular expressions. Results showed that in a mean of 134 (SD 9.7) EHRs, the six regular expressions could be matched. We did not use the pattern discovery method illustrated in this study because there was a semiautomatic step in the method. Repeating the pattern discovery method 1000 times would be time-consuming. A line graph was plotted to show five examples among all 1000 tests (Figure S6 in Multimedia Appendix 1).

**Table 1:** Six regular expressions based on linguistic patterns of the Chinese electronic health record corpus in this study.

Phenotype category and regular expressions		Example in Chinese (English translation)
**Phrase-based phenotypes**		
	re.compile^a^(“A^b^+P^c^(C^d^P)+”)	“无/A咳嗽/P、/C发热/P” (no cough or fever)
	re.compile(“AP+”)	“严重/A腹痛/P腹泻/P” (severe abdominal pain and diarrhea)
	re.compile(“A+P”)	“右下腹/A严重/A疼痛/P” (severe right-lower abdominal pain)
	re.compile(“A×PC×A+”)	“咳嗽/P，/C呈持续性/A” (cough, consistently)
**Logic-based phenotypes**		
	re.compile(“S^e^×L^f^N^g^U^h^”)	“白细胞/L 12×10^9^/N /L/U” (WBC^i^ 12 × 10^9^/L)
	re.compile(“S×LR^j^” )	“血/S糖/L升高/R” (high blood glucose)

^a^re.compile: a Python method used to compile a regular expression pattern.

^b^A: attribute.

^c^P: phenotype.

^d^C: punctuation.

^e^S: specimen.

^f^L: analyte.

^g^N: number.

^h^U: unit.

^i^WBC: white blood cell.

^j^R: results of laboratory examination.

### The Best Strategy for Recognizing PhenoSSU Instances

**Figure 5:**
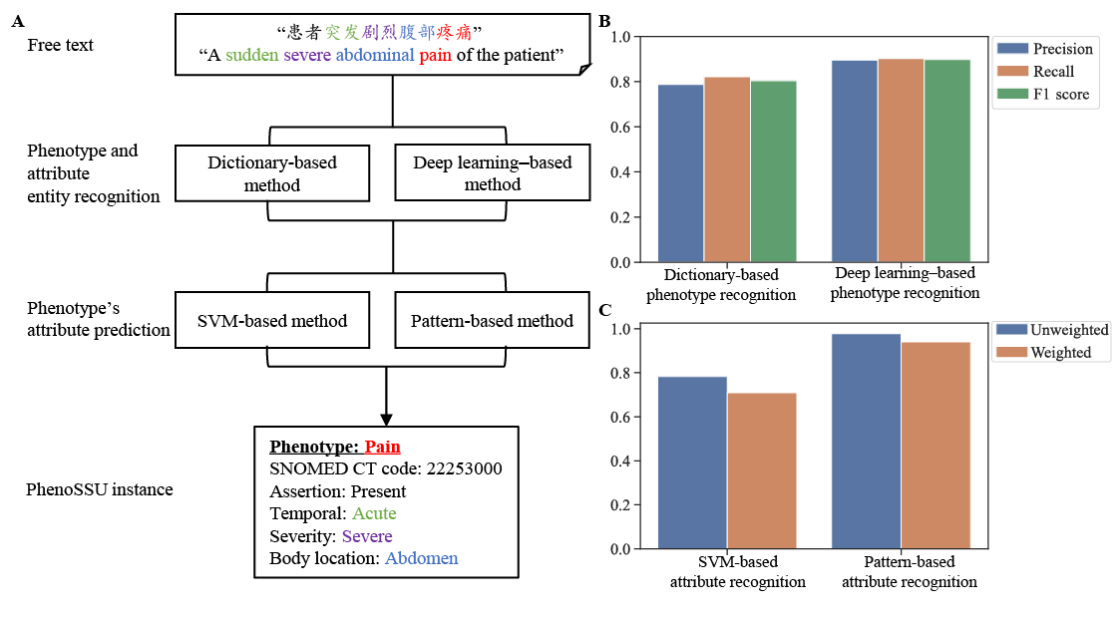
Determining the best strategy for recognizing PhenoSSU instances. A. The workflow of recognizing PhenoSSU instances from free text. B. The performance comparison between the dictionary-based method and the deep learning–based method in identifying phenotype concepts. C. The performance comparison between the SVM-based method and the pattern recognition–based method in recognizing a phenotype’s attributes. PhenoSSU: Semantic Structured Unit of Phenotypes; SNOMED CT: Systematized Nomenclature of Medicine–Clinical Terms; SVM: support vector machine.

Based on the linguistic patterns of Chinese EHRs, we developed a pattern recognition–based method to identify PhenoSSU instances. To find the best strategy for recognizing PhenoSSU instances, we developed and compared different methods in the subtasks of entity recognition and attribute prediction. The results in Figure 5 show that the best strategy was to recognize entities using the deep learning–based method and then predict the attribute values using the pattern recognition–based method.

Specifically, in the entity recognition subtask, the method of deep learning (ie, BERT-Bi-LTSM-CRF) achieved the best performance, with an F1 score of 0.898 (Figure 5, B). As a comparison, the dictionary-based method achieved an F1 score of 0.804. In the subtask of attribute prediction, the pattern recognition–based method had the best performance, with an accuracy of 0.977 and a weighted average of 0.940 (Figure 5, C). The SVM-based method achieved an accuracy of 0.783 and a weighted average of 0.709. The deep-phenotyping algorithm for Chinese EHRs had an overall accuracy of 0.844 on the test set. The detailed performances of the two models for predicting attribute values are shown in Table S4 in Multimedia Appendix 1.

### Case Study: Exploring the Real-World Evidence That Deep-Phenotyping EHRs Can Update Knowledge in Guidelines

With the pattern recognition algorithm, we could effectively structure phenotype information in Chinese EHRs. To demonstrate the potential application of deep phenotyping, a case study was conducted to update clinical guidelines by information retrieval of EHRs. In the case study, we selected the latest Chinese clinical guideline and 300 Chinese EHRs of chronic bronchitis. To recognize PhenoSSU instances from the guideline and the EHRs, we used the optimized hybrid strategy mentioned previously.

**Figure 6:**
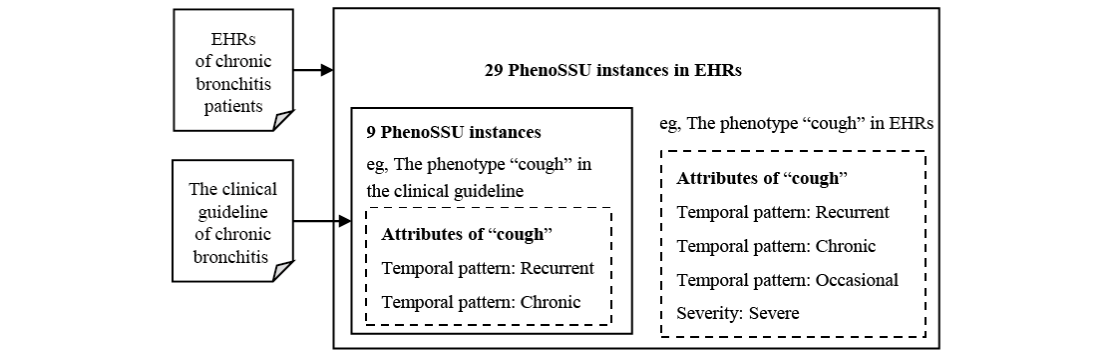
The comparison of PhenoSSU instances extracted from the clinical guidelines and electronic health records (EHRs) of chronic bronchitis. PhenoSSU: Semantic Structured Unit of Phenotypes.

A total of 9 and 29 PhenoSSU instances were identified from the clinical guideline and the EHRs of chronic bronchitis, respectively (details are shown in Tables S5-S7 in Multimedia Appendix 1). The 9 PhenoSSU instances identified in the clinical guideline appeared in the EHRs, which meant another 20 PhenoSSU instances in the EHRs were not covered in the clinical guideline. For example, “cough: chronic” and “cough: recurrent” both appeared in the clinical guideline and the EHRs. However, the current guideline could not give suggestions to accurately diagnose patients with occasional cough or severe cough as having chronic bronchitis (Figure 6). This real-world evidence hints at the feasibility of updating knowledge in clinical guidelines through deep phenotyping of large-scale EHRs.

## Discussion

### Principal Findings

In this study, we developed a simple but effective strategy to perform deep phenotyping of Chinese EHRs. The core of this strategy is learning linguistic patterns of PhenoSSU instances with a motif discovery tool from the field of bioinformatics. According to this research, biological sequence motif discovery tools could be used to effectively identify linguistic patterns of phenotype descriptions from medical texts after encoding them as DNA-like sequences. Meanwhile, the process of identifying linguistic patterns does not require too much annotation data; thus, our strategy is suitable for low-resource scenarios of deep-phenotyping Chinese EHRs.

This study was a preliminary attempt to use bioinformatics tools to tackle problems in medical informatics. By modeling natural language as single-letter sequences, it is possible that other advanced tools for analyzing biological sequences could also be used for processing natural language. For example, some researchers in the NLP field have applied a classic informatics algorithm, named the Basic Local Alignment Search Tool (BLAST), [33] to the text reuse detection task [34]. In Vesanto’s work [35], the 23 most-used English letters in the data set were calculated to form a simple one-to-one mapping between English letters and arbitrary amino acids. In this way, text was encoded into single-letter sequences that BLAST could handle to calculate similarities between texts. It is believed that future communications between bioinformatics and medical informatics will become more frequent [36].

It can be concluded from this study that there exist some regular linguistic patterns for phenotype narratives in Chinese EHRs. The origin of these linguistic patterns may be the common writing habits of clinicians who try to save time by recording clinical information faithfully in as few words as possible [37]. The reason our strategy does not require large annotation samples is that it uses the inner knowledge of linguistic patterns. As we know, data-hungry strategies, such as machine learning and deep learning, require many training samples to effectively identify patterns from data. However, there are many low-resource scenarios in practice that lack sufficient annotation samples for machine learning or deep learning. This is perhaps the reason why the majority (60%) of NLP studies in the medical domain have continued to use a knowledge-based approach rather than a machine learning–based approach [4]. In recent years, researchers have become increasingly focused on integrating machine learning with human knowledge [38], which is expected to become a new paradigm to deal with low-resource scenarios in medical informatics [39].

### Limitations

One limitation of this study was that linguistic patterns were learned from EHRs of respiratory diseases, which may not be applicable to other diseases. In addition, limited by the data size, the linguistic patterns in our study might be incomplete. In the future, we will continue to improve the algorithm with more Chinese EHRs from different hospital departments.

### Conclusions

We developed a simple but effective strategy to perform deep phenotyping of Chinese EHRs with limited fine-grained annotation data. Our work will promote the second use of Chinese EHRs and bring inspiration to other non–English-speaking countries.

## Appendix

Multimedia Appendix 1 Supplementary materials.

## Abbreviations

A: attribute (in the context of single-letter sequences)
BERT: Bidirectional Encoder Representations from Transformers
Bi-LSTM-CRF: bidirectional long short-term memory and conditional random field
BLAST: Basic Local Alignment Search Tool
C: punctuation (in the context of single-letter sequences)
CCKS: China Conference on Knowledge Graph and Semantic Computing
CEM: clinical element model
EHR: electronic health record
FHIR: Fast Healthcare Interoperability Resources
L: analyte (in the context of single-letter sequences)
LATTE: transforming lab test results
MEME: Multiple Expectation Maximums for Motif Elicitation
N: number (in the context of single-letter sequences)
NLP: natural language processing
O: other information (in the context of single-letter sequences)
P: phenotype (in the context of single-letter sequences)
PhenoSSU: Semantic Structured Unit of Phenotypes
R: results of laboratory examination (in the context of single-letter sequences)
S: specimen (in the context of single-letter sequences)
SemEval: Semantic Evaluation
SNOMED CT: Systematized Nomenclature of Medicine–Clinical Terms
SVM: support vector machine
U: unit (in the context of single-letter sequences)
WBC: white blood cell
