# Supplementary material for: Deep Phenotyping of Chinese Electronic Health Records by Recognizing Linguistic Patterns of Phenotypic Narratives With a Sequence Motif Discovery Tool: Algorithm Development and Validation
Source: J Med Internet Res. 2022 Jun 3;24(6):e37213. doi: 10.2196/37213 (PMC9206202; doi:10.2196/37213)
Supplement: Multimedia Appendix 1 [file jmir_v24i6e37213_app1.docx]

**Supplementary Materials**

Menu

[Supplementary Figures 2](#_Toc99029649)

[Figure S1. 2](#_Toc99029650)

[Figure S2. 2](#_Toc99029651)

[Figure S3. 3](#_Toc99029652)

[Figure S4. 3](#_Toc99029653)

[Figure S5. 4](#_Toc99029654)

[Figure S6. 4](#_Toc99029655)

[Supplementary Tables 5](#_Toc99029656)

[Table S1. 5](#_Toc99029657)

[Table S2. 6](#_Toc99029658)

[Table S3. 6](#_Toc99029659)

[Table S4. 6](#_Toc99029660)

[Table S5. 7](#_Toc99029661)

[Table S6. 7](#_Toc99029662)

[Table S7. 8](#_Toc99029663)

# Supplementary Figures

Figure S1. The composition of the PhenoSSU model.


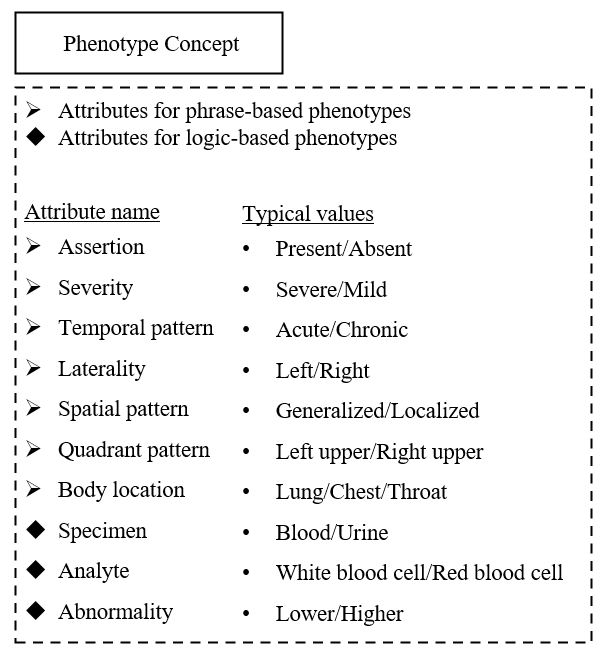


Figure S2. A snapshot of the knowledge base for laboratory tests.


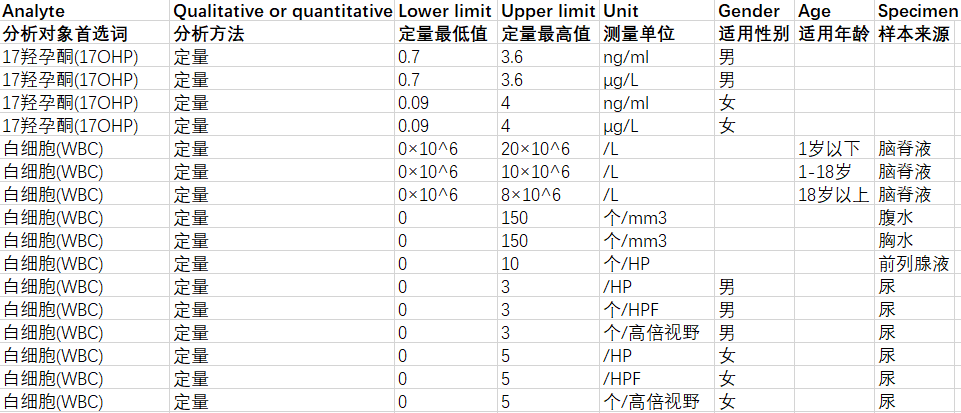


Figure S3. The mapping resource between Chinese phenotype terms and SNOMED-CT (A), including normalizing qualitative and quantitative test results (B).


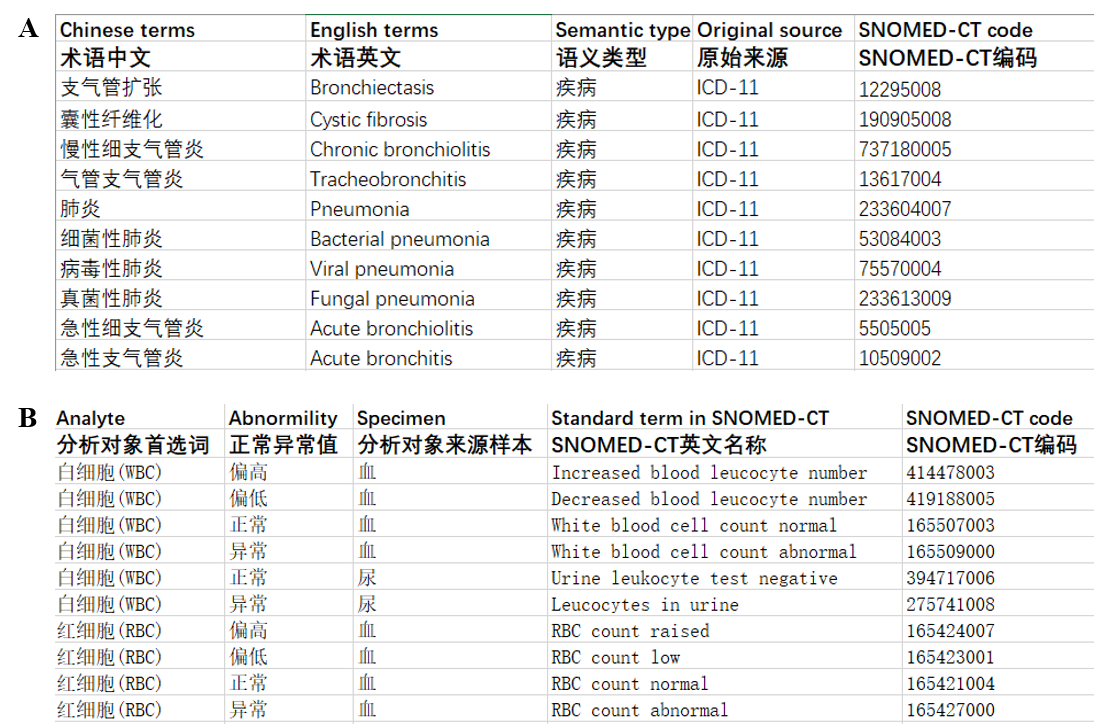


Figure S4. An example of position index recorded by the flashtext tool.


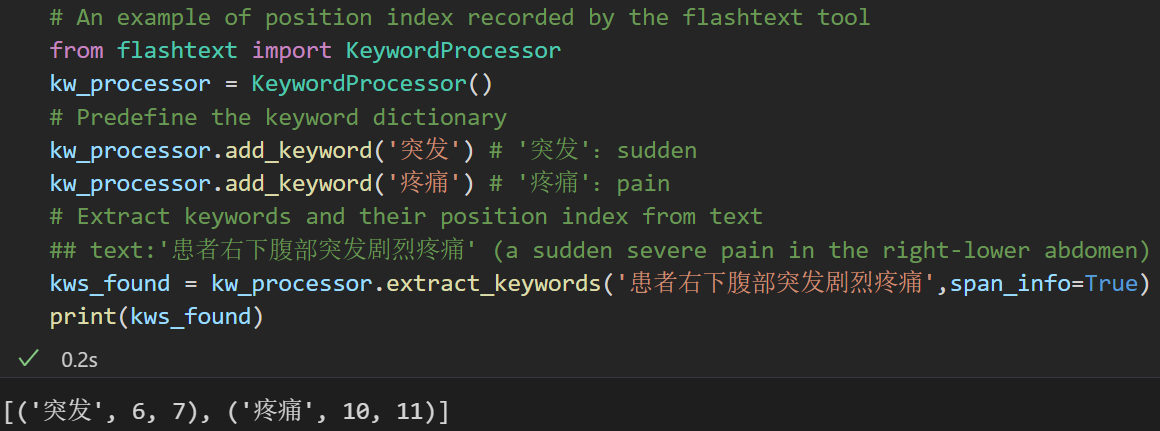


Figure S5. An illustration of the most significant motifs with different widths discovered by MEME motif discovery tool from the training set.


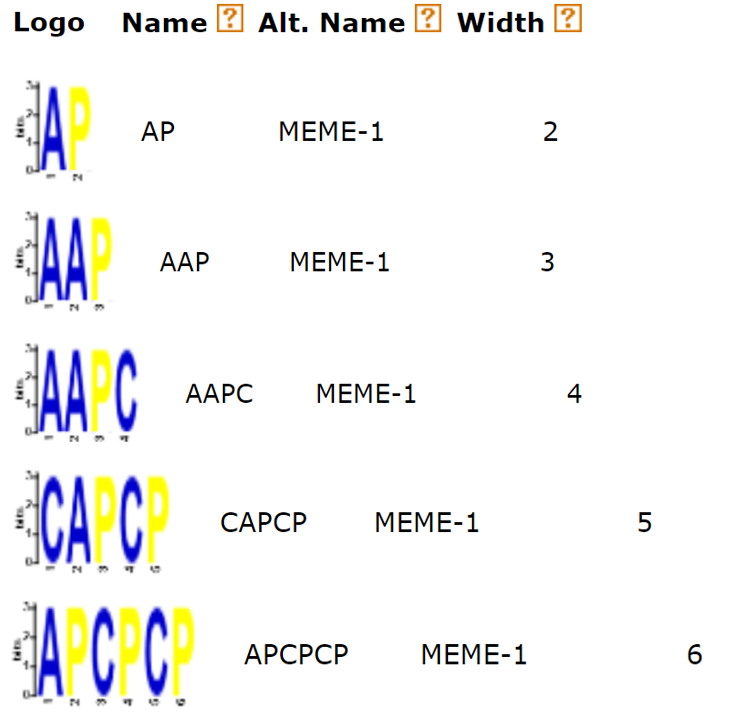


Figure S6. Five examples among the one thousand times test to find the minimum EHRs that could match all six regular expression learned in the study.


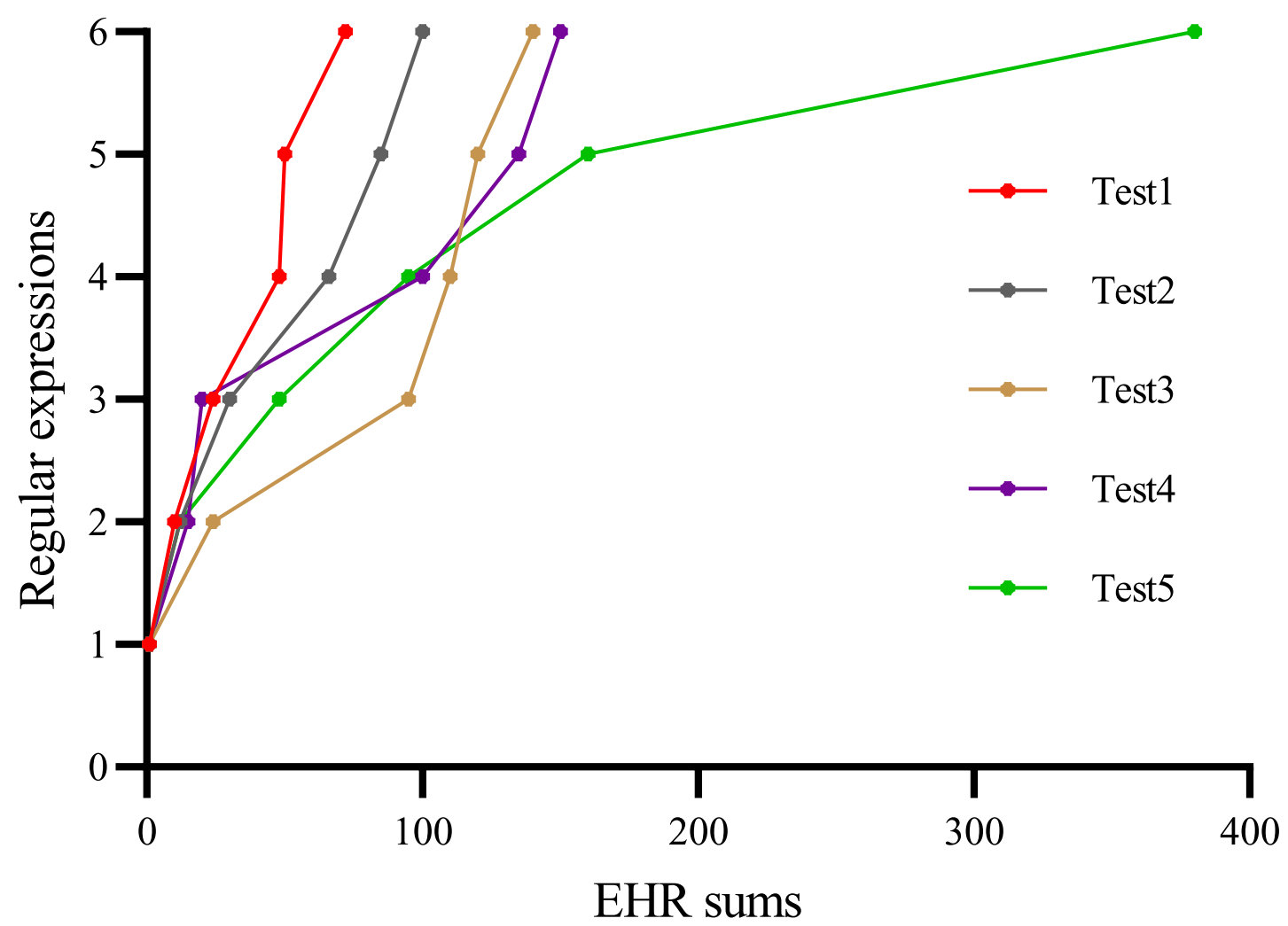


# Supplementary Tables

Table S1. The definition, typical values, and SNOMED-CT codes of attributes in the PhenoSSU model.

| Attribute | Definition | Typical values |
| --- | --- | --- |
| Assertion  (sct: 260245000) | Doctors’ belief status of patients’ medical problems. | Present (sct:52101004);  Absent (sct:2667000); |
| Severity  (sct: 272141005) | The intensity or degree of a manifestation. | Mild (sct: 255604002);  Moderate (sct: 6736007);  Severe (sct: 24484000); |
| Temporal pattern  (sct: 272103003) | The speed of disease development. | Acute (sct: 272118002);  Chronic (sct: 90734009);  Periodic (sct: 81591007);  Episodic (sct: 278499009);  Recurrent (sct: 255227004);  Prolonged (sct:255224006) |
| Spatial pattern  (sct: 255464007) | Patterns by which phenotypes affect one or more regions of the body. | Generalized (sct: 60132005);  Localized (sct: 255471002);  Diffuse (sct: 19648000); |
| Laterality  (sct: 272741003) | The localization of the specified phenotypic abnormality. | Left (sct: 7771000);  Right (sct: 24028007);  Unilateral (sct: 66459002);  Bilateral (sct: 51440002) |
| Quadrant pattern  (sct: 272137006) | The patterns by which phenotypes affect the four quadrants of the abdomen or thorax. | Left upper quadrant  (sct: 255481003);  Left lower quadrant  (sct: 255480002);  Right upper quadrant  (sct: 255497008);  Right lower quadrant  (sct: 255495000); |
| Specimen  (sct: 123038009) | A portion or quantity of material for use in testing, examination, or study. | Blood specimen  (sct: 119297000);  Urine specimen  (sct: 122575003);  Sputum specimen  (sct: 119334006); |
| Analyte  (sct: 272524002) | Substances to be analyzed in testing, examination, or study. | White blood cell  (sct: 767002);  Red blood cell  (sct: 14089001); |

Table S2. Knowledge base of phenotype terms.

| Phenotype term resource | Source of phenotype term resource |
| --- | --- |
| ICD-10, Chinese version | National Health Commission of the PRC |
| ICD-11, Chinese version | National Health Commission of the PRC |
| HPO, Chinese version | https://www.chinahpo.net/chpo/ |

Table S3. Regular expressions and specific linguistic patterns in the corpus.

| Regular expression | Specific linguistic pattern | Total |
| --- | --- | --- |
| **re.compile(‘A+P’)** |  | 921 |
|  | AP | 748 |
|  | AAP | 129 |
|  | Others | 44 |
| **re.compile(‘AP+’)** |  | 267 |
|  | APP | 166 |
|  | APPP | 70 |
|  | Others | 31 |
| **re.compile(‘A+P(CP)+’)** |  | 294 |
|  | APCP | 208 |
|  | APCPCP | 67 |
|  | Others | 19 |
| **re.compile(‘A*PC*A+’)** |  | 53 |
|  | PA | 37 |
|  | Others | 16 |
| **re.compile(‘S*LNU’)** |  | 233 |
|  | LNU | 182 |
|  | SLNU | 51 |
| **re.compile(‘S*LR’ )** |  | 43 |
|  | LR | 27 |
|  | SLR | 16 |

Table S4. The weighted accuracy (WA) of pattern-based and SVM-based method for predicting attribute values on the test set.

| Attributes | Pattern Recognition (WA) | SVM (WA) |
| --- | --- | --- |
| Assertion | 0.969 | 0.785 |
| Severity | 0.902 | 0.710 |
| Temporal pattern | 0.933 | 0.699 |
| Laterality pattern | 0.848 | 0.640 |
| Quadrant pattern | 0.786 | 0.577 |
| Spatial pattern | 0.807 | 0.601 |
| Body location | 0.912 | 0.635 |
| Total | 0.940 | 0.709 |

Table S5. The PhenoSSU instances of phrase-based phenotype descriptions identified from 300 Chinese EHRs of chronic bronchitis.

| Phenotype concept | Attribute | Frequency |
| --- | --- | --- |
| **Cough** |  |  |
|  | recurrent | 37 |
|  | chronic | 24 |
|  | severe | 17 |
|  | occasional | 12 |
| **Expectoration** |  |  |
|  | recurrent | 23 |
|  | chronic | 15 |
| **Emphysema** | possible | 10 |
| **Pant** |  |  |
|  | possible | 10 |
|  | chronic | 18 |
|  | mild | 10 |
| **Congestion** | throat | 20 |
| **Bloody sputum** | occasional | 12 |
| **Wheezing rale** | mild | 20 |
| **Limb weakness** | right | 12 |
| **Chest tightness** | chronic | 13 |
| **Cardiopalmus** | recurrent | 12 |
| **Edema** | lower limbs | 27 |

Table S6. The PhenoSSU instances of logic-based phenotype descriptions identified from 300 Chinese EHRs of chronic bronchitis.

| Analyte | Abnormality | Frequency |
| --- | --- | --- |
| **Red blood cell** |  |  |
|  | lower | 14 |
|  | higher | 31 |
| **White blood cell** | higher | 68 |
| **Breathing rate** | higher | 13 |
| **Lymphocyte** | lower | 43 |
| **Pulse rate** | higher | 37 |
| **Body temperature** | higher | 49 |
| **Heart rate** | higher | 28 |
| **Hemoglobin** |  |  |
|  | higher | 30 |
|  | lower | 31 |
| **Blood pressure** | higher | 45 |
| **Neutrophils** | higher | 47 |

Table S7. The PhenoSSU instances identified from the clinical guideline of chronic bronchitis.

| Phenotype concept | Attribute |
| --- | --- |
| **Cough** |  |
|  | recurrent |
|  | chronic |
| **Expectoration** |  |
|  | recurrent |
|  | chronic |
| **Bloody sputum** | occasional |
| **Wheezing rale** | mild |
| **Emphysema** | possible |
| **Pant** |  |
|  | possible |
|  | chronic |
